# Stark County Medical Society

**Published:** 1860-09

**Authors:** J. S. Pashley

**Affiliations:** Secretary


### STARK COUNTY MEDICAL SOCIETY.

At a meeting of the physicians of Stark county, called for the purpose of discussing the propriety of organizing a Medical Society for the county, held at the office of Dr. R. Boardman, in Elmira, on Saturday, August 11, 1860, the following were present:

Elmira—Dr. E. R. Boardman, and J. G. Boardman, medical student.

Bradford—Drs. S. T. C. Washburn, and J. G. Lamper.

Wyoming—Dr. William Hayden.

Osceola—Dr. J. S. Pashley.

Dr. Lamper was called to the Chair, and Dr. Boardman chosen Secretary.

A motion prevailed to organize an Association to be known as the Stark County Medical Society.

The following were chosen as permanent officers of the Society:

President—Dr. E. R. Boardman.

Secretary—Dr. J. S. Pashley.

Treasurer—Dr. Wm. Hayden.

Censors—Drs. J. G. Lamper, S. T. C. Washburn, William Hayden.

On motion, the Code of Ethics of the National Medical Association was adopted for the government of the Society.

The following persons were appointed as a committee to prepare Constitution, By-Laws, and Fee Bill for the Society, to report at the next meeting: Drs. Boardman, Pashley, and Lamper.

On motion, Jas. G. Boardman was admitted to membership as a medical student.

The Secretary was directed to extend an invitation to all regular physicians in the county, with their students, to unite and co-operate with the Society.

On motion, the plan of raising necessary funds by equal assessment was adopted.

Dr. E. R. Boardman, of Elmira was appointed to deliver an address at the next meeting.

The Secretary was directed to furnish copies of the proceedings, of the meeting, to the *Chicago Medical Journal* and *Stark County News*, for publication.

Adjourned to meet at Wyoming, on Monday, September 3d, 1860, at 10 A. M.

J. S. Pashley, Secretary.

